# *ENO2* knock-out mutants in *Arabidopsis* modify the regulation of the gene expression response to NaCl stress

**DOI:** 10.1007/s11033-018-4292-7

**Published:** 2018-08-17

**Authors:** Chao Chen, Yonghua Zhang, Pan Ye, Xiaofeng Ma, Chaoxing Zheng, Genfa Zhang

**Affiliations:** 1 The Laboratory of Vector Biology and Control, College of Engineering, Beijing Normal University (Zhuhai), Zhuhai, People’s Republic of China; 2 Beijing Key Laboratory of Gene Resource and Molecular Development, College of Life Sciences, Beijing Normal University, Beijing, People’s Republic of China

**Keywords:** *Arabidopsis*, Mutant *eno2*, NaCl stress, Gene expression

## Abstract

There is a growing awareness that some dual-function enzymes may provide a directly evidence that metabolism could feed into the regulation of gene expression via metabolic enzymes. However, the mechanism by which metabolic enzymes control gene expression to optimize plant stress responses remains largely unknown *i*n *Arabidopsis thaliana. LOS2*/*ENO2* is a bifunctional gene transcribed a functional RNA that translates a full-length version of the ENO2 protein and a truncated version of the MBP-1 protein. Here, we report that *eno2* negatively regulates plant tolerance to salinity stress. NaCl treatment caused the death of the mutant *eno2*/*eno2* homozygote earlier than the wild type (WT) *Arabidopsis*. To understand the mechanism by which the mutant *eno2* had a lower NaCl tolerance, an analysis of the expressed sequence tag (EST) dataset from the WT and mutant *eno2 Arabidopsis* was conducted. Firstly, the most identified up- and down-regulated genes are senescence-associated gene 12 (*SAG12*) and isochorismate mutase-related gene, which are associated with salicylic acid (SA) inducible plant senescence and endogenous SA synthesis, respectively. Secondly, the differentially regulated by salt stress genes in mutant *eno2* are largely enriched Gene Ontology(GO) terms associated with various kinds of response to stimulations. Thirdly, in the Kyoto Encyclopedia of Genes and Genomes (KEGG) mapping, we find that knocking out *ENO2*-influenced genes were most enriched into metabolite synthesis with extra plant-pathogen interaction pathway and plant hormone signal transduction pathway. Briefly, with the translation shifting function, *LOS2*/*ENO2* not only influenced the genes involved in SA synthesis and transduction, but also influenced genes that participate in metabolite synthesis in cytoplasm and gene expression variation in nuclear under salt stress.

**Electronic supplementary material:**

The online version of this article (10.1007/s11033-018-4292-7) contains supplementary material, which is available to authorized users.

## Introduction

In *Arabidopsis, LOS2* (low expression of osmotically responsive genes 2)/*ENO2* (Enolase 2) is a bifunctional gene. In addition to coding for the full-length ENO2, which catalyzes a key step in glycolysis, it can be alternatively translated (from the second start codon) to a truncated protein, c-MYC binding protein (MBP-1), which is a regulator involved in abscisic acid responses [1]. Recently, Eremina *et al*. argued that MBP-1 could repress the accumulation of *LOS2*/*ENO2* transcription through a negative feedback mechanism [2]. Moreover, *LOS2*/*ENO2* was suggested to be a cold-specific transcriptional repressor [3] that is required for salt stress responses [4]. It is generally accepted that plants actively respond to salt stress by reprogramming their whole metabolism or alternating signal pathways to enhance stress tolerance [5, 6]. The molecular mechanism of *ENO2* associated with the salinity tolerance in tonoplasts by *Arabidopsis* enolase mutant *los2* is well demonstrated by Barkla *et al*. [4]. However, the mechanism by which the nuclear or cytoplasm isoforms of enolase controls the response of *Arabidopsis* to salinity stress is still unclear.

Finding the upstream or downstream effector genes of *LOS2*/*ENO2* will significantly contribute to the understanding on the mechanism by which metabolic enzymes directly control the gene expression. The 454 GS FLX sequencing platform provides a rapid method for analyzing the different transcriptomes of the WT and mutant *eno2*, especially in plants with the *eno2*/*eno2* homozygote for which EST sequences are not currently available for salinity stress. In this work, a mutant *eno2* homozygote was identified from two salt-treated seedling EST libraries in which WT and mutant *eno2* were sequenced. In all, 2,735 up-regulated and 2,318 down-regulated genes in mutant *eno2* were identified. Two SA-associated genes, *SAG12* and isochorismate mutase-related, were identified as the most up- and down-regulated genes, respectively. It indicates that *LOS2*/*ENO2* is possible to negatively regulates plant tolerance to salinity stress through SA signaling pathway. Using GO and KEGG-based pathway analysis, we show that the differentially regulated by salt stress genes in mutant *eno2* are enriched into the response to stimulations GO terms, but mostly are enriched in metabolite synthesis pathway. High-throughput analysis and the characterization of modulated genes would provide a foundation for detailed studies on the genetic connections between *LOS2*/*ENO2* activity and the *Arabidopsis* response to salinity stress at the posttranscriptional level.

## Materials and methods

### Confirmation of homozygous *ENO2* T-DNA Insertion Lines

The T-DNA mutagenized *Arabidopsis* (Columbia-0 ecotype) for *ENO2* (SALK_021737, AT2G36530) was confirmed by Beijing Key Laboratory of Gene Resource and Molecular Development. For *ENO2* fragment analysis, the primers 5′-AATGGATGTTGCCGCTTCAGAGTTC-3′ and 5′-TAAGTCAGCAATGAATGTGTCCTCG-3′ were used. Additionally, the primers of the *Arabidopsis* housekeeping gene actin-2 (At3g18780) were used as follows: 5′-TAACTCTCCCGCTATGTATGTCGCC-3′ and 5′-TTTCTGTGAACGATTCCTGGACCTG-3′. The PCR program was as follows: denaturation at 94 °C for 5 min; 30 cycles 94 °C for 30 s, 46 °C for 30 s, and 72 °C for 1 min; the 72 °C extension step lasts approximately 10 min. Then, the size of amplified products was analyzed by ethidium bromide staining in agarose gels.

### Plant growth

For growth measurements, 4-day-old seedlings of WT and mutant *eno2 Arabidopsis* were grown on an MS culture [0.7% (w/v) agar] medium containing 3% (w/v) sucrose and transferred to soil in a climate chamber (16 h light and 8 h dark at 21 °C for 3 weeks). Whole plants were assayed for resistance to 300 mM of NaCl at various time points using WT and homozygous plants for 18 days. For the gene expression profiling assay, 4-day-old seedlings were also transferred to identical plates with the MS culture. After 6 days of culture, the plants were rinsed briefly with the MS solution and further cultured in the MS medium containing 300 mM NaCl for 24 h. The seedlings were harvested at the 24 h time points for NaCl treatment and were frozen in liquid nitrogen before the start of the sequence experiments.

### RNA extraction and cDNA library construction

The total RNA of the whole plants was prepared using EasyPure® Plant RNA Kit (TransGen, Beijing, China), After a DNase treatment, approximately 2.0 µg of poly(A)^+^ RNA were isolated from the total RNA using the Oligotex mRNA Mini Kit (Qiagen, Chatsworth, CA). One µg of mRNA was mixed with 10 pmol 3′ SMART CDS Primer II A (BD Clontech, PaloAlto, CA), incubated at 70 °CC for 2 min and then quenched on ice for 2 min. One µl of PowerScript RT with the first strand buffer and dNTPs were added, and the reaction was incubated at 42 °C for 1 h. The first strand of cDNA (5 µl) was then used as a template for the second strand polymerase chain reaction with 2 PCR Primer II A in a 100 µl reaction. The PCR program was as follows: 95 °C for 1 min; 15 cycles of 95 °C for 5 s, 65 °C for 5 s; and 68 °C for 6 min. Amplified cDNA was purified with the PureLink PCR Purification Kit (Invitrogen, Paisley, UK). The resulting cDNA was fragmented and sequenced on a 454 GS FLX Titanium platform (454 Life Sciences, Roche, Branford, CT).

### Sequence assembly

DNA sequencing was performed on a 454 GS FLX Titanium Sequencing System according to the manufacturer’s instructions. The cDNA libraries, which ranged from 300 to 800 bp (base pair), were added to the FLX specific adapters (Adapter A and B) and then nebulized and selected for denaturation to generate single-stranded DNA. Approximately 5 µg of single-stranded DNA were amplified by emulsion PCR before sequencing on the 454 GS FLX platform.

The length of the DNA sequencing reads in the dataset is approximately 200 bp (Supplementary Fig. S1). The raw sequencing data stored in the BAM file format contains low-quality sequences and adaptor sequences that were not suitable for the gene scan. First, reads shorter than 30 bp after pre-processing were excluded. Then, after trimming the adapter reads, if the length was less than the length of the set threshold, the reads were also excluded. The raw data were then converted to clean data, which were used for subsequent data analysis.

### Unigene function annotation, GO classification, and metabolic pathway analysis

Supplementary Table S1 illustrates the summary from the mapping results (mapping to reference genes). There were 25,582 unigenes identified in WT plants and 26,357 unigenes identified in mutant *eno2* under NaCl stress. A total of 17,454,311 (66.13%) and 17,003,457 (65.64%) unique matches in each dataset were obtained. The unigenes were annotated by a BLAST search against GO (http://www.geneontology.org), NCBI GenBank (http://www.ncbi.nlm.nih.gov/), and KEGG (http://www.genome.jp/kegg) databases. The transcripts were assigned a GO term based on the top blast hits in all retrieve queries, and the differentially expressed unigenes were classified into GO categories under the major categories of biological process. We also used BLASTX against the KEGG database, which is a resource for understanding the high-level functions and utilities of the biological system, to assign the differentially expressed unigenes to special metabolic pathways that represent molecular interactions and reaction networks.

## Results and discussion

### Characterization of *eno2* mutants

**Fig. 1:**
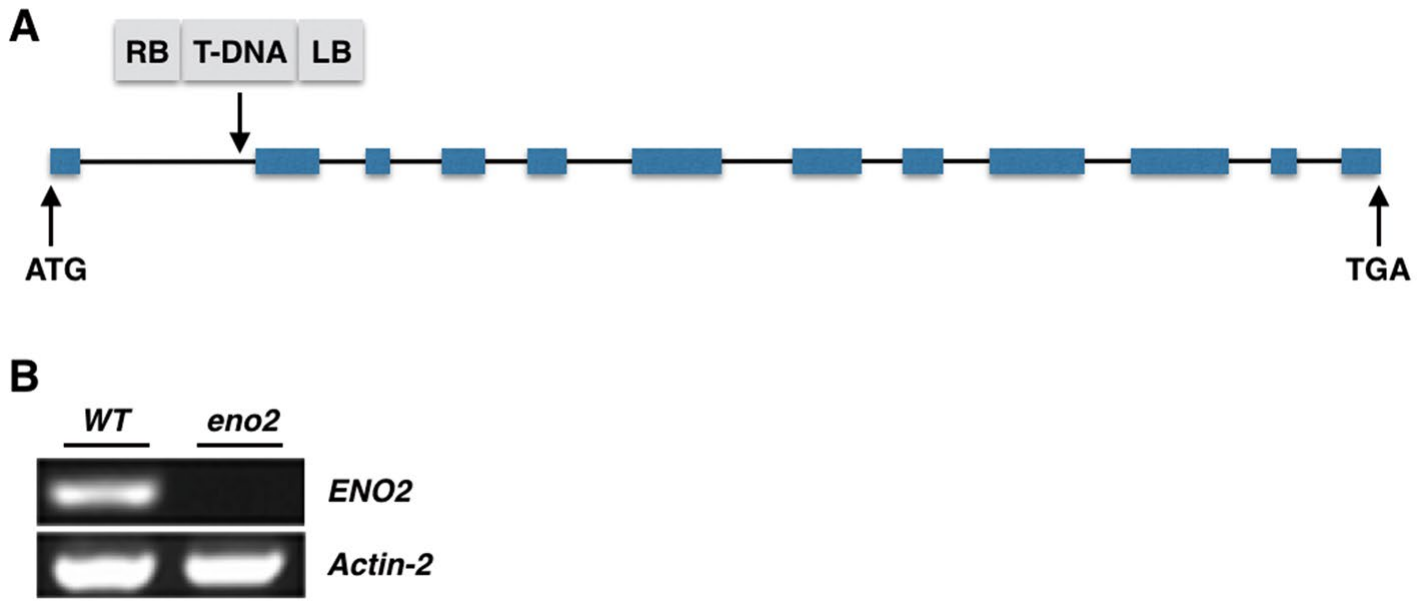
Localization of T-DNA insertion and expression variation of *ENO2*. **a** Localization of T-DNA. The down-arrow demonstrates the orientation of the inserted T-DNA in the *ENO2* gene. RB and LB show the right and left T-DNA border, respectively. Black lines indicate introns and filled bars indicate exons. **b** RT-PCR of ENO2 gene in WT plants and *eno2* mutants. Actin-2 indicates the housekeeping gene as a control

The T-DNA integration position was at the first intron of the *ENO2* gene (Fig. 1a), and our results show that the RT-PCR fragments of the *ENO2* transcripts from the mutant *eno2* did not produce a signal (Fig. 1b). While the introns are nucleotide sequences that are removed by RNA splicing during maturation, they are integral to the regulation of gene expression. In some cases, they are actually engaged in the regulation of gene expression, such as through nonsense mediated decay [7] and mRNA export [8]. In this work, the broken integrity of the first intron leads to the silencing of the *ENO2* gene, indicating that the first intron plays an essential role in *ENO2* gene expression.

### T-DNA insertion of *ENO2* results in decreased NaCl tolerance in *Arabidopsis*

**Fig. 2:**
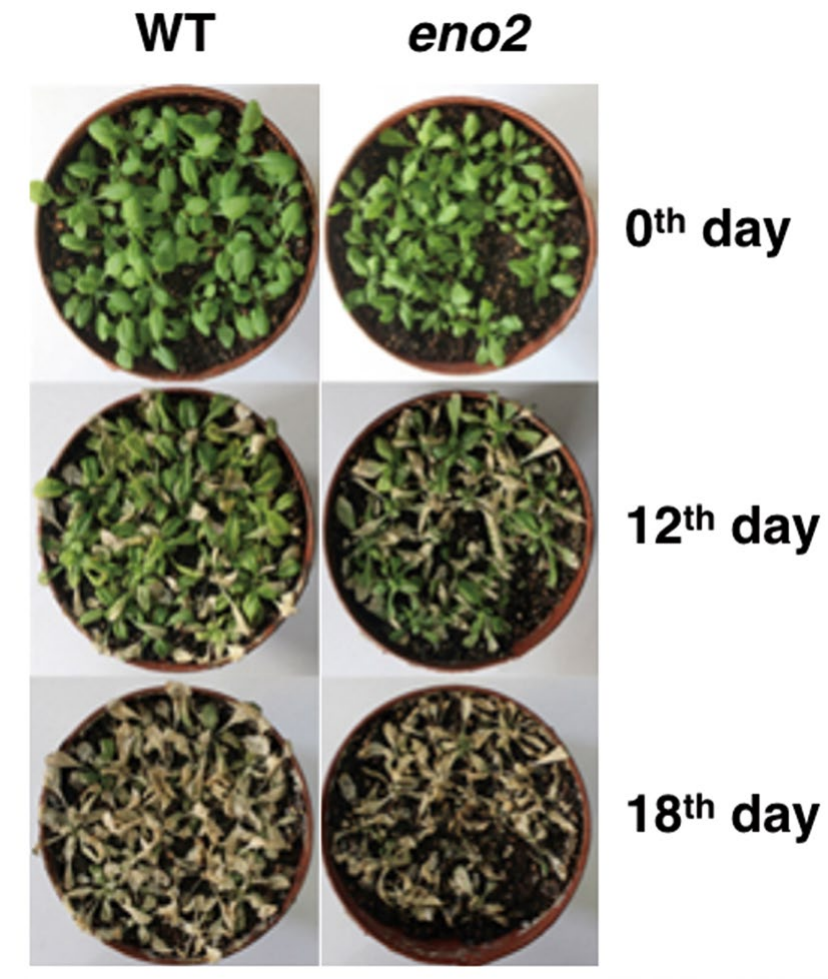
Decreased resistance of eno2 mutant to NaCl stress. Four-week-old WT and *eno2* plants were treated with 300 mM NaCl. Phenotypes were observed at 0th (on the day prior to the commencement of the salt stress treatment), 12th, and 18th day

As NaCl stress activates many signaling pathways, we compared the phenotypes of T-DNA inserted mutant *eno2* and WT *Arabidopsis* under 300 mM NaCl stress. Each experiment was repeated three times, and at least 40 lines of mutant *eno2* or WT plants were treated for each time. In visual appearance, mutant *eno2* subjected to NaCl stress display no obviously fewer green leaves than WT plants on the 12th day after the salt treatment (Fig. 2, a randomly selected sample). Although the glycolytic enzymes, such as ENO2, that play an important role in regulating salt stress were widely studied [9, 10], little attention has been paid to the mechanism of the knock-out of *ENO2* that results in damage and apoptosis of leaves in salt treatment. The physiological modulation of the plant response to salinity relies on the proteins involved in signaling, changes in gene expression, and protein metabolism. The gene expression profile of these two *Arabidopsis* lines with different salinity tolerance should be investigated at the transcriptional level to understand the variation between the mutant *eno2* and WT plants.

### Differentially expressed genes analysis

**Fig. 3:**
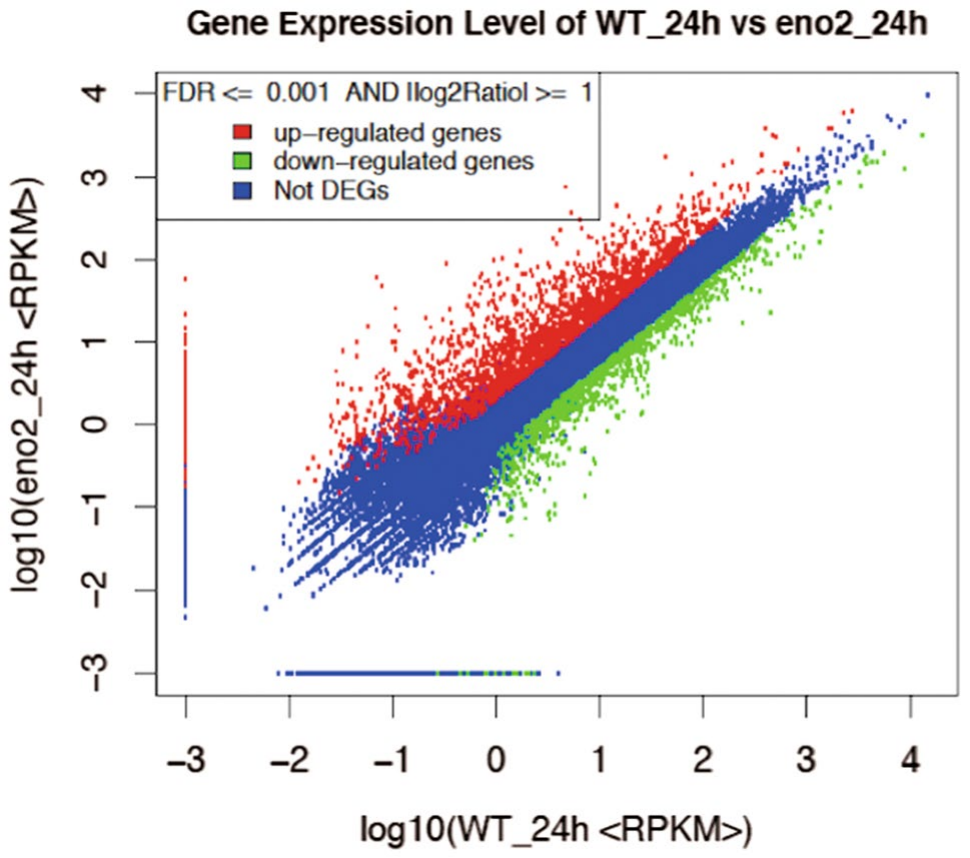
Differentially expressed genes (DEGs) analysis of WT and *eno2* mutant libraries. X-axis shows the log10 value of WT plant RPKM, and Y-axis represents the log10 value of *eno2* mutant. The red and green dots denote the significantly different expression in each dataset (two-fold change), whereas the blue dot indicates no significant difference in the expression of the genes

The RPKM (reads per kb per million reads) method was used to measure gene expression levels. Differentially expressed genes (DEGs) with high abundance and differential expression exhibited between the two genotypes were the focus of this work. With NaCl treatment, there were 2302 up-regulated genes and 1126 down-regulated genes with the parameters set as log2 (fold change), ratio ≥ 1 and FDR (false discovery rate) ≤ 0.001(Fig. 3).

The most up-regulated gene was AT5G45890.1, *SAG12*, (log2 ratio: 57.05, gi:18422605), which encodes a cysteine protease influenced by cytokinin, auxin, and sugars. The *SAG12* gene can be induced by WRKY53, which is a SA inducible transcription factor that promotes plant senescence [11, 12]. Here, we suggest that silencing of *ENO2* directly leads to high expression of *SAG12*, which may play a crucial role in *eno2* entering the senescence phase (yellow) earlier than WT under salt stress.

Alternatively, the most down-regulated gene is AT1G35513.1, isochorismate mutase-related gene (log2 ratio: − 11.19, gi:297839345). This gene may translate a hypothetical protein that influences the conversion of chorismate to prephenate and then to the products phenylalanine and tyrosine. The isochorismate pathway is the main source of endogenous SA synthesized in chloroplast rather than in the cytoplasm, where the SA is synthesized by the phenylalanine ammonia-lyase pathway [13, 14]. The mutant of the SA induction deficient two (*sid2*) gene in *Arabidopsis* down-regulates the expression of the isochorismate synthase (*ICS1*) gene, leading to hypersensitivity to salt stress [15, 16]. Hence, the suppression of isochorismate mutase-related SA biosynthesis by ENO2 may be an essential factor for salt tolerance in *Arabidopsis*.

### GO annotations of differentially expressed genes analysis

To explain all observed gene expression changes for the process that *eno2* negatively regulates plant tolerance to salinity stress, GO analysis should be taken in this work. With the corrected *p* value sorted in ascending order, there are only 12 genes annotated in regulation of response to biotic stimulus and 6 genes in cellular response to abiotic stimulus. But we confirmed that the response to abiotic stimulus and response to abiotic stimulus GO terms, known as a response to salt stress, are classified into the top 10 enriched GO terms with high significance (Table 1). Nevertheless, all GO terms in top 10 are associated with the response to stimulus.

**Table 1:** The process ontology terms of DEGs with corrected *p* value less than 0.05

Gene ontology term	Cluster frequency	Genome frequency of use	Corrected *p* value
Response to stimulus	1111 out of 2215 genes, 50.2%	7620 out of 20,969 genes, 36.3%	4.57E−42
Response to stress	654 out of 2215 genes, 29.5%	3859 out of 20,969 genes, 18.4%	7.47E−39
Defense response	238 out of 2215 genes, 10.7%	1057 out of 20,969 genes, 5.0%	3.85E−28
Response to oxygen-containing compound	189 out of 2215 genes, 8.5%	789 out of 20,969 genes, 3.8%	2.91E−25
Response to organic substance	390 out of 2215 genes, 17.6%	2330 out of 20,969 genes, 11.1%	2.44E−19
Response to biotic stimulus	207 out of 2215 genes, 9.3%	1020 out of 20,969 genes, 4.9%	3.54E−18
Response to carbohydrate stimulus	115 out of 2215 genes, 5.2%	436 out of 20,969 genes, 2.1%	4.60E−18
Response to abiotic stimulus	388 out of 2215 genes, 17.5%	2403 out of 20,969 genes, 11.5%	2.21E−16
Response to chemical stimulus	520 out of 2215 genes, 23.5%	3517 out of 20,969 genes, 16.8%	5.58E−15
Response to other organism	177 out of 2215 genes, 8.0%	901 out of 20,969 genes, 4.3%	9.42E−14
Response to endogenous stimulus	318 out of 2215 genes, 14.4%	2013 out of 20,969 genes, 9.6%	1.64E−11
Multi-organism process	193 out of 2215 genes, 8.7%	1091 out of 20,969 genes, 5.2%	1.84E−10
Secondary metabolic process	108 out of 2215 genes, 4.9%	525 out of 20,969 genes, 2.5%	6.00E−09
Secondary metabolite biosynthetic process	80 out of 2215 genes, 3.6%	356 out of 20,969 genes, 1.7%	3.92E−08
Response to hormone stimulus	282 out of 2215 genes, 12.7%	1864 out of 20,969 genes, 8.9%	9.05E−08
Signaling	299 out of 2215 genes, 13.5%	2078 out of 20,969 genes, 9.9%	5.39E−06
Phenylpropanoid biosynthetic process	58 out of 2215 genes, 2.6%	252 out of 20,969 genes, 1.2%	7.25E−06
Phenylpropanoid metabolic process	64 out of 2215 genes, 2.9%	293 out of 20,969 genes, 1.4%	1.10E−05
Cell death	70 out of 2215 genes, 3.2%	336 out of 20,969 genes, 1.6%	1.81E−05
Death	70 out of 2215 genes, 3.2%	336 out of 20,969 genes, 1.6%	1.81E−05
Response to reactive oxygen species	31 out of 2215 genes, 1.4%	100 out of 20,969 genes, 0.5%	1.83E−05
Single-organism biosynthetic process	127 out of 2215 genes, 5.7%	744 out of 20,969 genes, 3.5%	2.60E−05
Flavonoid biosynthetic process	33 out of 2215 genes, 1.5%	113 out of 20,969 genes, 0.5%	3.32E−05
Ketone biosynthetic process	35 out of 2215 genes, 1.6%	125 out of 20,969 genes, 0.6%	4.21E−05
Response to oxidative stress	37 out of 2215 genes, 1.7%	137 out of 20,969 genes, 0.7%	4.96E−05
Response to osmotic stress	153 out of 2215 genes, 6.9%	965 out of 20,969 genes, 4.6%	0.00014
Flavonoid metabolic process	33 out of 2215 genes, 1.5%	121 out of 20,969 genes, 0.6%	0.00019
Programmed cell death	58 out of 2215 genes, 2.6%	291 out of 20,969 genes, 1.4%	0.00132
Cellular ketone metabolic process	35 out of 2215 genes, 1.6%	144 out of 20,969 genes, 0.7%	0.0017
Immune system process	59 out of 2215 genes, 2.7%	304 out of 20,969 genes, 1.4%	0.00264
Monocarboxylic acid metabolic process	92 out of 2215 genes, 4.2%	543 out of 20,969 genes, 2.6%	0.00294
Cell wall modification	29 out of 2215 genes, 1.3%	112 out of 20,969 genes, 0.5%	0.00333
Defense response to fungus	34 out of 2215 genes, 1.5%	142 out of 20,969 genes, 0.7%	0.00341
Response to fungus	34 out of 2215 genes, 1.5%	144 out of 20,969 genes, 0.7%	0.00474
Jasmonic acid metabolic process	16 out of 2215 genes, 0.7%	44 out of 20,969 genes, 0.2%	0.0049
Response to light intensity	31 out of 2215 genes, 1.4%	130 out of 20,969 genes, 0.6%	0.00982
Immune effector process	17 out of 2215 genes, 0.8%	52 out of 20,969 genes, 0.2%	0.01302
Response to radiation	147 out of 2215 genes, 6.6%	1003 out of 20,969 genes, 4.8%	0.02057
Cell wall organization	41 out of 2215 genes, 1.9%	201 out of 20,969 genes, 1.0%	0.02502
Response to water deprivation	21 out of 2215 genes, 0.9%	79 out of 20,969 genes, 0.4%	0.04666

Except the most up- or down-regulated genes, there may be some other well-characterized salt response genes shows no obviously significance in *p* value. In this work, we find that the response to salt stress GO terms contain 8 DEGs as follows: *AT3G21370.1, AT1G05675.1, AT5G60270.1, AT5G63990.1, AT5G19000.2, AT5G64000.1, AT1G05680.1 and AT5G09290.1*. Among of this, *AT1G05675.1* and *AT1G05680.1* are also classified into the hyperosmotic salinity response GO term (Table 2). These two GO terms statistical differences are not significant, but the genes annotated into these terms are highly impacted the plant salinity tolerance. The regulation of these well-characterized salt response genes provides a valuable framework that directly links *LOS2*/*ENO2* to the salt response functions.

**Table 2:** Differentially expressed salinity tolerance related genes

Process Ontology term	GeneID	WT_24 h-RPKM	eno2_24 h-RPKM	log2 Ratio	FDR	Description
Response to salt stress/hyperosmotic salinity response	AT1G05680.1	7.901282047	85.60965475	3.437614841	0	Symbols: UGT74E2 | Uridine diphosphate glycosyltransferase 74E2
Response to salt stress	AT5G64000.1	4.637864696	21.66939811	2.224126445	7.91E−54	Symbols: SAL2, ATSAL2 | Inositol monophosphatase family protein
Response to salt stress	AT5G60270.1	1.156503206	7.482285588	2.693709767	7.27E−44	Symbols: LECRK-I.7 | Concanavalin A-like lectin protein kinase family protein
Response to salt stress	AT5G09290.1	1.708224293	6.512534541	1.930721703	1.48E−13	Symbols: | Inositol monophosphatase family protein
Response to salt stress/hyperosmotic salinity response	AT1G05675.1	0.237445936	1.87277171	2.979504076	1.73E−08	Symbols: | UDP-Glycosyltransferase superfamily protein
Response to salt stress	AT3G21370.1	0.781954259	0.001	− 9.610940408	2.13E−07	Symbols: BGLU19 | beta glucosidase 19
response to salt stress	AT5G63990.1	0.965002378	2.707512504	1.488363598	4.85E−05	Symbols: | Inositol monophosphatase family protein
Response to salt stress	AT5G19000.2	1.836643156	0.606054045	− 1.599552992	8.21E−05	Symbols: BPM1 | BTB-POZ and MATH domain 1

### KEGG pathways of differentially expressed genes analysis

**Fig. 4:**
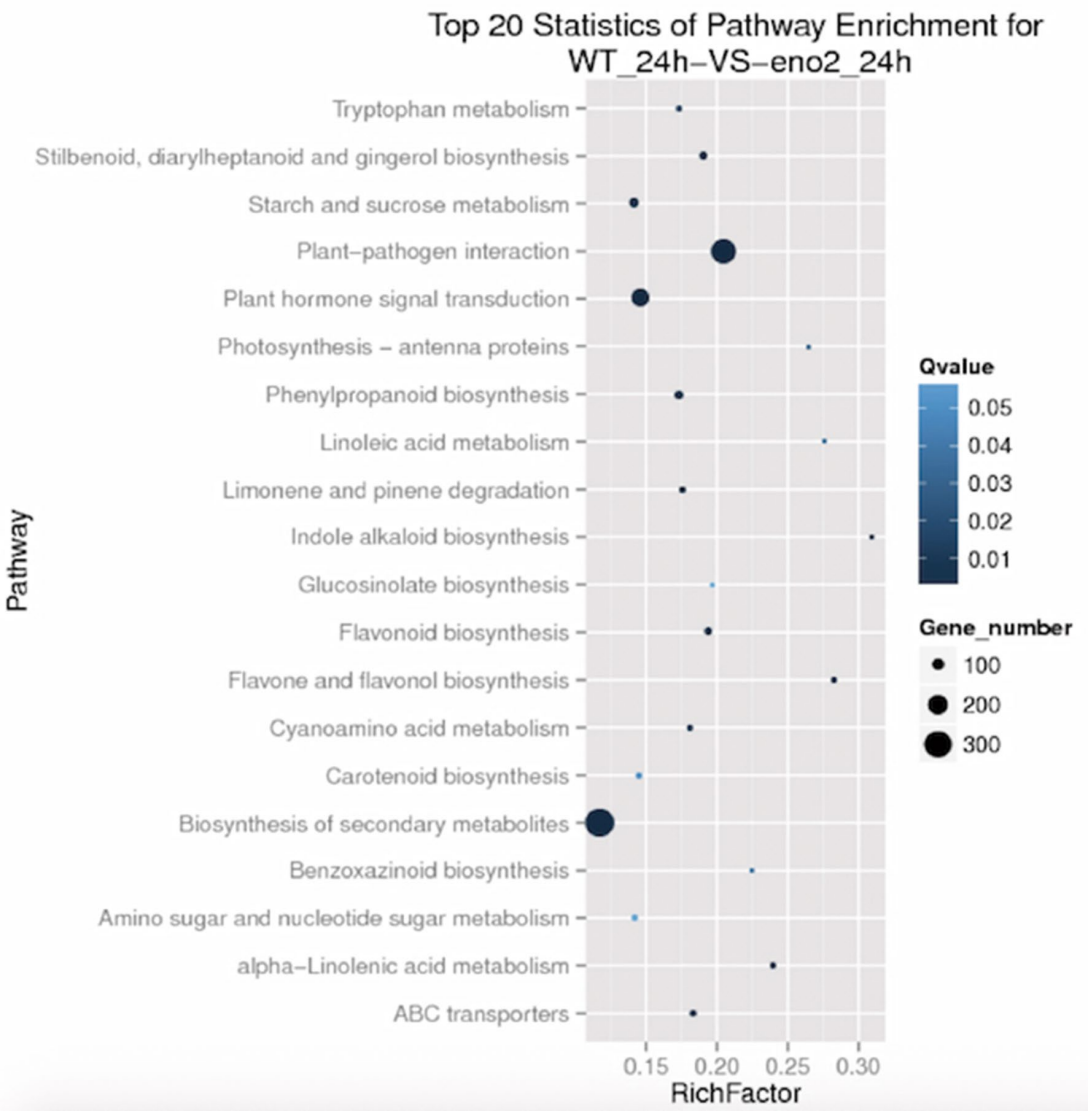
Scatter plot of KEGG pathway enrichment statistics. The RichFactor refers to the ratio generated from dividing DEG numbers parsed in a special pathway by the total number of genes parsed in the same pathway. Greater RichFactor means greater intensiveness. Q-value is the corrected *p* value ranging from 0 to 1; lower values mean greater intensiveness. The figure demonstrates the enrichment degree of the top 20 entries to the pathway

KEGG analysis provides a bioinformatics resource for linking genomes to life and the environment. Pathway enrichment analyses identify significantly enriched metabolic pathways or signal transduction pathways in DEGs. To identify the biological pathways that were changed by the *ENO2* mutation, up- and down-regulated genes were assigned to KEGG pathways with enrichment statistics (Fig. 4). The top five enrichment pathways (sorted by q-value) were plant-pathogen interaction (KO04626), plant hormone signal transduction (KO04075), flavone and flavonol biosynthesis (KO00944), stilbenoid, diarylheptanoid and gingerol biosynthesis (KO00945), and phenylpropanoid biosynthesis (KO00940). The pathways with the highest gene count numbers were sorted as follows: the biosynthesis of secondary metabolites with 321 DEGs, plant-pathogen interaction with 263 DEGs, plant hormone signal transduction with 174 DEGs, starch and sucrose metabolism with 70 DEGs, and phenylpropanoid biosynthesis with 64 DEGs. Unfortunately, only 11 DEGs appeared in the glycolysis pathway while considering ENO2 as a key step in the catalytic process. These results suggest that the decreasing response of the mutant *eno2* to NaCl may be due to the role of MBP-1 in the nucleus rather than the role of enolase in glycolysis. Paradoxically, the top 20 statistics of enrichment of pathway have 17 metabolic pathways. Consequently, the re-routing of conversion of 2-phosphoglycerate (2-PG) to phosphoenolpyruvate (PEP) is expected to interfere with other metabolic processes to adapt to salt tolerance.

There were 263 DEGs identified in the plant-pathogen interaction pathway and 174 DEGs identified in the plant hormone signal transduction pathway. Ten transcription factors (sometimes called sequence-specific DNA-binding factors) were identified in these obviously changed pathways. WRKY25, WRKY29, and MYC2 were identified in the plant-pathogen interaction pathway. ARF, B-ARR, ABF, ERF1/2, BZR1/2, MYC2, and TGA were identified in the plant hormone signal transduction pathway. The variation of these ten genes, which directly target DNA, may control the flow of genetic information from DNA to mRNA along with MBP-1 (a truncated ENO2 localized in the nucleus) and is responsible for the expression of stress-activated genes related to *Arabidopsis* tolerance and adaptation.

## Conclusion

The evaluation of this bifunctional metabolism enzyme in salt stress has been extremely difficult to study, especially as one mRNA generates with two proteins. Our study generated gene expression data for WT and mutant *eno2*, increasing the available knowledge on how the *ENO2* gene is associated with NaCl stress. First, we identified *SAG12*, a transcription factor that promotes plant senescence, which established the direct relationship between the *ENO2* gene and the senescence process. Up-regulated expression of *SAG12* consistent with our previous observations in phenotypes of mutant *eno2*. Additionally, the most down-regulated gene, which leads to hypersensitivity to salt stress, was also identified in this work. Using GO and KEGG of DEGs analysis, we find that the GO terms show more genes response to stimulations terms and KEGG shows more metabolite synthesis pathways. This phenomenon could be rationally explained with the bifunctional molecule of *ENO2* gene. Finally, in the process of *eno2* mutants adaptation or tolerance to salinity stress, the significant regulated eight well-characterized salt response genes and ten transcription factors (belongs to plant-pathogen interaction and hormone signal transduction pathway) were identified in this work.

## Electronic supplementary material

Below is the link to the electronic supplementary material.

Supplementary **Figure S1**. Sequence length distribution of *Arabidopsis* 454 ESTs and GenBank ESTs. The red line shows WT plants; the blue line shows *eno2* mutant. Y-axis: count number; X-axis: size in bp. (TIF 1840 KB)

Supplementary material 2 (DOCX 41 KB)
