# Supplementary figures and images for: ENO2 knock-out mutants in Arabidopsis modify the regulation of the gene expression response to NaCl stress
Source: Mol Biol Rep. 2018 Aug 17;45(5):1331–8. doi: 10.1007/s11033-018-4292-7 (PMC6156758; doi:10.1007/s11033-018-4292-7)

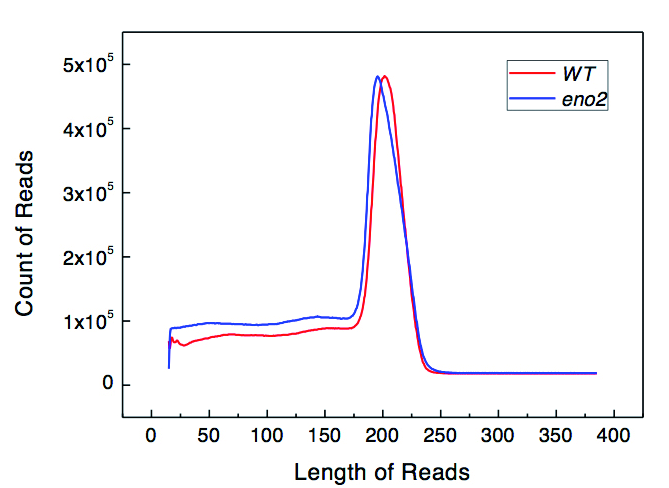

Supplement: Supplementary file 1 — Supplementary Figure S1. Sequence length distribution of Arabidopsis 454 ESTs and GenBank ESTs. The red line shows WT plants; the blue line shows eno2 mutant. Y-axis: count number; X-axis: size in bp. (TIF 1840 KB) [file 11033_2018_4292_MOESM1_ESM.tif]
