# Supplementary material for: ENO2 knock-out mutants in Arabidopsis modify the regulation of the gene expression response to NaCl stress
Source: Mol Biol Rep. 2018 Aug 17;45(5):1331–8. doi: 10.1007/s11033-018-4292-7 (PMC6156758; doi:10.1007/s11033-018-4292-7)
Supplement: Supplementary file 2 — Supplementary material 2 (DOCX 41 KB) [file 11033_2018_4292_MOESM2_ESM.docx]

| **Supplement Table S1. Summary of mapping result to reference genes.** | | | | | |  |
| --- | --- | --- | --- | --- | --- | --- |
| **Sample ID** | **Total Reads** | **Total Base Pairs** | **Total Mapped Reads** | **Perfect Match** | **Mismatch** | **Unique Match** |
| WT_24h | 26,394,414 | 4,242,673,891 | 26,195,839 | 13,774,039 | 12,421,800 | 17,454,311 |
|  | -100.00% | -100.00% | -99.25% | -52.19% | -47.06% | -66.13% |
| eno2_24h | 25,903,887 | 4,181,071,083 | 25,663,466 | 12,792,700 | 12,870,766 | 17,003,457 |
|  | -100.00% | -100.00% | -99.07% | -49.39% | -49.69% | -65.64% |
